# Multimodal MRI insights into glymphatic clearance and blood-brain barrier integrity in healthy aging

**DOI:** 10.1162/IMAG.a.1391

**Published:** 2026-10-08

**Authors:** Anjan Bhattarai, Yufei D. Zhu, Barah Albuhwailah, Pauline Maillard, Charles DeCarli, Audrey P. Fan

**Affiliations:** Department of Neurology, University of California, Davis, Davis, CA, United States; Department of Biomedical Engineering, University of California, Davis, Davis, CA, United States; Stevens Neuroimaging and Informatics Institute, University of Southern California, Los Angeles, CA, United States

**Keywords:** aging, glymphatic system, arterial spin labeling, blood–brain barrier, water exchange rate, diffusion tensor imaging, DTI-ALPS, free water

## Abstract

Aging is associated with impaired CSF clearance in preclinical models, but its impact on blood-brain barrier (BBB) health and glymphatic function in humans remains unclear. The BBB and glymphatic functions are complementary and may partially overlap, yet their interaction is yet to be fully elucidated. In this study, we aimed to compare BBB integrity, extracellular free water, and glymphatic function between healthy young and older adults, and to examine mechanistic relationships among these measures using multimodal MRI techniques. 30 healthy participants with no known neurological or psychiatric disorders, including 12 younger (26 ± 3 years) and 18 older individuals (75 ± 7 years), underwent MRI scans on a 3T Siemens Prisma scanner. Multimodal MRI techniques were used, including diffusion-prepared arterial spin labeling to measure the water exchange rate (Kw) across the blood-brain barrier (BBB); free water (FW) imaging to quantify extracellular free water in white matter; and diffusion tensor imaging along perivascular spaces (DTI-ALPS) to assess glymphatic function. These measures were compared between younger and older individuals. Mediation analysis was performed to assess the mechanistic relationship between BBB integrity and glymphatic function and to test whether this relationship was mediated by free water. Older individuals showed reduced whole-brain Kw (p = 0.021), increased white matter FW (p = 0.002), and reduced DTI-ALPS (p < 0.001) compared to younger adults. Kw was positively associated with DTI-ALPS (β = 0.402 × 10^−2^, p = 0.022) and negatively associated with FW (β = -0.676 × 10^−3^, p = 0.008). DTI-ALPS was negatively associated with FW (β = -3.487, p = 0.001). Significantly reduced Kw was also observed in the cortical grey matter of older adults. Reduced Kw may reflect impaired BBB function, while reduced DTI-ALPS and increased FW indicate impaired glymphatic clearance. FW mediated the relationship between Kw and DTI-ALPS (95% CI [0.00014, 0.00404], p = 0.035), suggesting a potential mechanistic link between BBB function, extracellular water content, and glymphatic clearance efficiency. Overall, this study provides novel multimodal MRI insights into BBB and glymphatic alterations in healthy aging and may inform the development of MRI biomarkers to preserve cognitive health.

## 1 Introduction

Healthy brain aging is a complex process which may involve subtle alterations in vascular, metabolic, and clearance pathways that maintain cerebral homeostasis (Banks et al., 2021). One such pathway is the glymphatic system, which is defined as a brain-wide waste clearance system that facilitates the removal of interstitial solutes and metabolic waste, including β-amyloid (Aβ) and tau, from the brain tissue (Iliff et al., 2012; Verheggen et al., 2018). This system was first described in 2012 based on fluid transport pathways in mice (Iliff et al., 2012). It includes the flow of subarachnoid cerebrospinal fluid (CSF) into the brain’s interstitial spaces through para-arterial influx. Once inside the brain parenchyma, CSF mixes with interstitial fluid (ISF), which is then cleared through major veins (i.e., para-venous routes). This exchange is supported by the perivascular spaces and regulated by the neurovascular unit (NVU). The NVU includes components such as blood vessels, the blood-brain barrier (BBB) formed by endothelial cells and connected by tight junctions, perivascular spaces that are essential for CSF flow, and astrocytes that express aquaporin-4 (AQP4) at their endfeet (Iliff et al., 2012; Rowsthorn et al., 2023). These AQP4 water channels facilitate convective movement of CSF into interstitial spaces. The BBB protects the brain against ion fluctuations in the bloodstream and helps maintain optimal ISF conditions to preserve homeostasis (Verheggen et al., 2018). The BBB and glymphatic functions are thus complementary and may partially overlap, but their interaction is yet to be fully elucidated.

Impaired glymphatic clearance can be an initiating factor of age-related brain pathologies (Verheggen et al., 2018). Preclinical studies indicate that glymphatic function declines with advancing age. In mouse models, impaired glymphatic function in the aging brain slows the clearance of interstitial Aβ, increasing the risk of developing neurodegenerative diseases (Kress et al., 2014). Glymphatic dysfunction has also been associated with altered AQP4 expression, and changes in CSF influx, which depend on arterial pulsatility (Kress et al., 2014; Mestre et al., 2018). In parallel, increased BBB permeability has been reported in cognitively unimpaired older individuals (Verheggen et al., 2020). Additionally, altered BBB permeability has been reported in the aging human hippocampus (Montagne et al., 2015). BBB transport and glymphatic clearance are complementary mechanisms involved in removing interstitial solutes and maintaining fluid homeostasis (Verheggen et al., 2018). However, the mechanistic relationship between these functions in human aging has been poorly understood. Understanding this link could pave the way for developing MRI biomarkers to support cognitive health in aging populations.

Recent advances in MRI now allow us to assess BBB integrity and glymphatic function in vivo. Diffusion-prepared arterial spin labeling (DP-ASL), a non-contrast MRI technique, allows us to estimate the water exchange rate (Kw) across the BBB as a surrogate measure of BBB health (Shao et al., 2019). Kw has been associated with a diffusion tensor imaging (DTI)-based extracellular free water (FW) measure in older adults, indicating its role in CSF clearance (Pappas et al., 2024). Recent multimodal neuroimaging findings further indicate relationships between neurovascular integrity and fluid transport, including associations between FW and enlarged perivascular spaces, as well as between BBB Kw and cerebral blood flow (CBF) (Rowsthorn et al., 2025). The mechanistic link between BBB Kw, FW, and glymphatic function remains unclear, providing the motivation for this study to investigate their contribution to glymphatic clearance in aging. The diffusion weighted imaging (DWI)-based technique, diffusion tensor imaging along the perivascular pathway (DTI-ALPS), provides a proxy for glymphatic function in the brain (Taoka et al., 2017). Lower DTI-ALPS index values have been associated with reduced diffusivity along perivascular spaces and interpreted as indicative of impaired glymphatic function (Taoka et al., 2017). In this study, we aimed to compare BBB integrity, white matter free water, and glymphatic function between healthy young and older adults, and to assess the associations among these measures using multimodal MRI techniques, including DP-ASL, DTI-ALPS, and FW. Based on prior evidence demonstrating an association between BBB water exchange and white matter free water (Pappas et al., 2024), together with the role of glymphatic pathways in brain fluid homeostasis (Hablitz & Nedergaard, 2021), we hypothesized that aging-related alterations in BBB function may be associated with increased white matter free water accumulation and altered glymphatic function. These analyses may provide novel insights into glymphatic clearance and may guide interventions to preserve cognitive health in aging populations*.*

## 2 Methods

Ethics approval for this study was obtained from the University of California, Davis (UC Davis) Institutional Review Board. The study was conducted in accordance with the Belmont Report. All participants were at least 18 years old and provided written informed consent. Older participants were recruited through the UC Davis Alzheimer’s Disease Research Center (ADRC) longitudinal cohort and were at least 55 years of age. Older participants were evaluated to be cognitively normal within 12 months of the MRI scan through clinical evaluation by experienced neurologists at UC Davis Health and had no history of neurological disorder. Healthy, younger participants were recruited from an institutional registry of study volunteers.

### 2.1 MRI data acquisition

30 healthy participants with no known neurological or psychiatric disorders, including 12 (8 females, 4 males) younger (26 ± 3 years) and 18 (13 females, 5 males) older individuals (75 ± 7 years), underwent MRI scans on a 3T Siemens Prisma scanner using a 32-channel head coil. DWI, DP-ASL, and structural MRI data were acquired and processed as in Figure 1. Data were acquired with the following acquisition parameters: DWI with voxel size = 2 x 2 x 2 mm^3^, 127 volumes at b-values = 0, 500, 1000, and 2000 s/mm². DP-ASL was acquired using a 3D gradient-and-spin-echo (GRASE) readout pseudo-continuous arterial spin labeling (PCASL) to estimate Kw (TR = 4000 ms, TE = 36.5 ms, field of view = 224 mm, matrix size = 64 × 64, 12 slices, voxel size = 3.5 × 3.5 × 8 mm³, labeling duration = 1500 ms, post-label delay = 1800 ms) (Zhu et al., 2025). DP-ASL acquisition included 20 repetitions with diffusion weightings (b-value) at 0 and 50s/mm^2^. A flow-encoding arterial spin tagging (FEAST) (Wang et al., 2003) acquisition (b = 0 and 14 s/mm²) was also performed to estimate arterial transit time (ATT). T1-weighted (T1W) magnetization-prepared rapid gradient-echo (MPRAGE) structural images were acquired with a voxel size of 1.0 × 1.0 × 1.0 mm³.

**Fig. 1.**
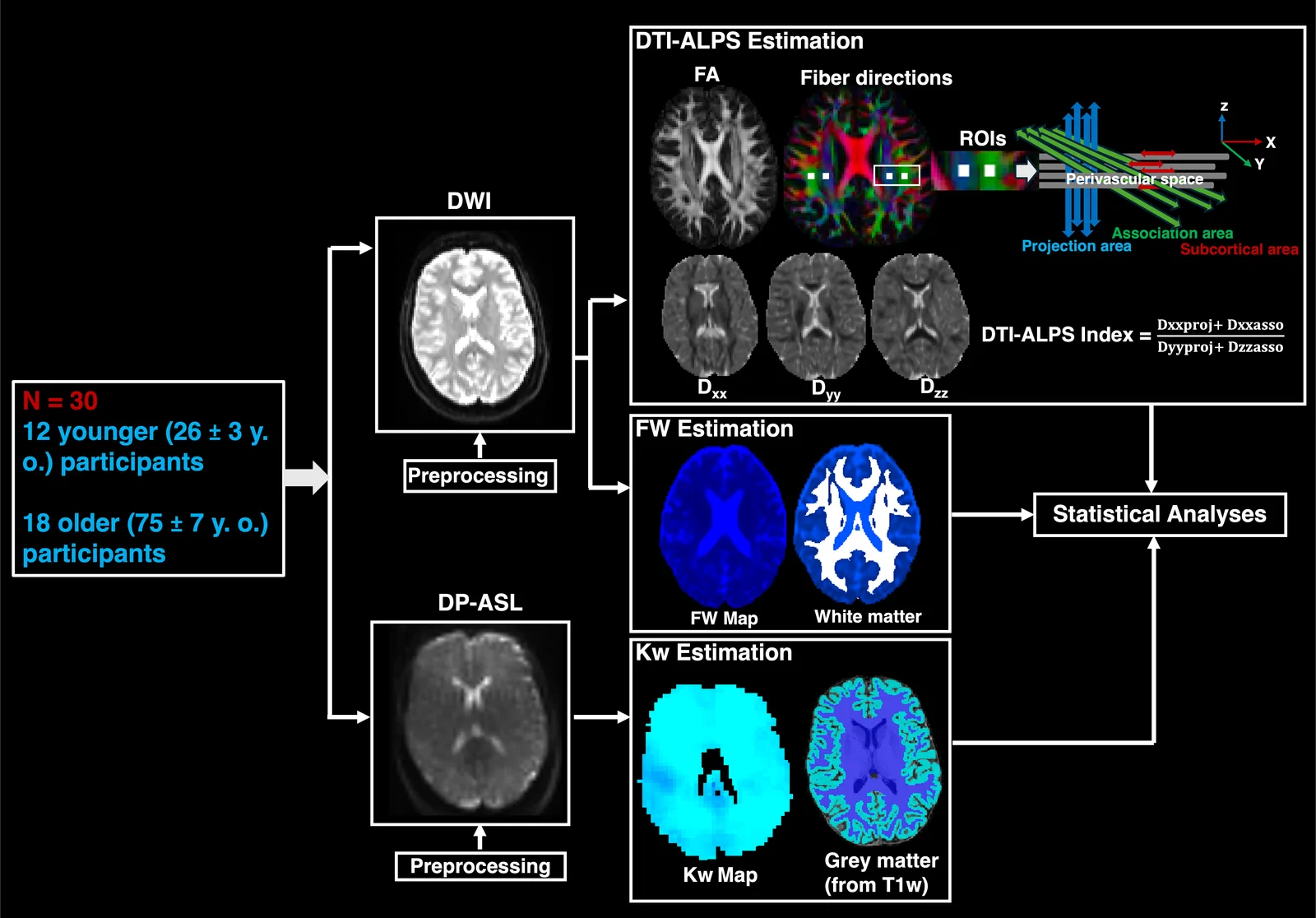
Summary of workflow and data processing. DWI: Diffusion-weighted imaging; DP-ASL: Diffusion-prepared arterial spin labeling; DTI-ALPS: Diffusion tensor imaging–analysis along the perivascular space; FA: Fractional anisotropy; Dxx, Dyy, Dzz: Diffusion coefficients; Kw: Water exchange rate.

### 2.2 DP-ASL data processing

Raw DP-ASL data were processed to generate individual Kw images using the water exchange quantification (WEQ) toolbox with a two-compartment kinetic model, with ATT used in the Kw estimation, as previously described (Shao et al., 2019; Zhu et al., 2025). In addition, CBF maps and global mean CBF values were generated from the same DP-ASL acquisition and included as exploratory analyses. The grey matter regions of interests (ROIs) were obtained using FreeSurfer cortical parcellation of the T1W images (Fischl, 2012). Kw was estimated for whole-brain, cortical grey matter, hippocampus, entorhinal cortex, and amygdala, with voxel-wise values averaged to obtain mean Kw (bilateral averaging applied to all regions except whole-brain). Whole-brain Kw was used as the primary measure (Shao et al., 2024). Cortical grey matter and regions including the hippocampus, entorhinal cortex, and amygdala were included as exploratory analyses, given the known involvement of these regions in aging-related processes (Bhattarai et al., 2025; Mormino et al., 2021).

### 2.3 DWI data processing

DWI images were preprocessed using MRtrix3 and FSL softwares (Jenkinson et al., 2012; Tournier et al., 2019). Preprocessing steps included denoising (dwidenoise), Gibbs ringing correction (mrdegibbs), motion and eddy-current correction with dwipreproc (FSL eddy, using topup for susceptibility-induced distortion correction), and bias field correction (dwibiascorrect) (Tournier et al., 2019).The preprocessed data were then used to fit diffusion tensors with FSL’s dtifit. ROIs (square, 3 × 3 voxels, 6 × 6 mm²) (X. Zhang et al., 2023) were manually placed in the projection and association areas along the perivascular space, guided by color-coded fractional anisotropy maps, and carefully inspected for anatomical accuracy. Bilateral individual diffusion coefficients along x, y, and z directions in the projection and association fibers and DTI-ALPS measures were estimated for each participant. Left and right DTI-ALPS measures were computed separately and then averaged to obtain a single ALPS index for each participant. The ALPS index was computed as:

$ALPS\;index\;=\frac{\left(Dxx_{proj}\;+\;Dxx_{assoc}\right)}{\left(Dyy_{proj}+\;Dzz_{assoc}\right)}$; where Dxx, Dyy, and Dzz are the diffusion coefficients along the x, y, and z directions in the projection (proj) and association (asso) fiber ROIs, following the approach of Taoka et al. (2017).

Preprocessed DWI data were modeled using a two-compartment bi-tensor approach, which separates the diffusion signal into tissue and isotropic free water contributions. This yielded voxelwise FW maps, reflecting extracellular water content (Maillard et al., 2022; Pasternak et al., 2009). FW maps were generated using MarkVCID2 Biomarker Kit Protocols (https://markvcid.partners.org/markvcid2-protocols-resources). FSL’s FAST was used to segment T1-weighted images to obtain whole-brain white matter masks (Y. Zhang et al., 2001). Mean FW values were computed within the whole-brain white matter mask for each participant. As an exploratory analysis, regional free-water (FW) values were extracted from nine white matter tracts using the Johns Hopkins University (JHU ICBM) atlas in MNI space (Mori et al., 2008), and their associations with Kw were assessed. Tract details and analyses are provided in Supplementary Table S1.

### 2.4 Statistical analysis

Subjects were grouped by age (younger vs. older adults). Statistical comparisons between groups were performed using two-sample t-tests. Degrees of freedom, p-values, t-statistics, and 95% confidence intervals were calculated and reported. Group differences in Kw were assessed across the whole brain and in selected regions of interest (cortical grey matter, hippocampus, entorhinal cortex, and amygdala), with ROI-level analyses presented as exploratory.

Similarly, group differences in DTI-ALPS index, individual diffusion coefficients, and white matter FW fraction were computed. Pearson linear correlation was used to examine the relationship between DTI-ALPS index and whole-brain white matter FW fraction. Linear regression analyses were then performed to assess whether whole-brain Kw were associated with FW fraction and DTI-ALPS index. Exploratory analyses were performed to evaluate potential sex effects on the imaging measures Kw, DTI-ALPS index and FW fraction using linear regression, with age group and sex as covariates. Tract-specific FW values and their associations with Kw were assessed using linear regression.

A mediation analysis was conducted in MATLAB to test whether whole-brain Kw is associated with DTI-ALPS, mediated by FW (i.e., to assess whether FW explains part of this association). In an exploratory mediation analysis, lateral ventricular volume residualized for estimated total intracranial volume was included as a covariate to account for the potential effects of brain atrophy, following the approach described previously (Pappas et al., 2024). Statistical significance of the mediation effect was assessed using a bootstrapping approach with 5,000 simulations to compute 95% confidence intervals. All analyses were performed in MATLAB.

## 3 Results

### 3.1 DTI-ALPS

We observed reduced DTI-ALPS index in older individuals compared to younger individuals (p < 0.001) (Table 1). Group differences were also evident in individual diffusion coefficients, with a significantly increased diffusion coefficient along the y-axis in the projection fibers (D_yy_proj) (p = 0.043) in older participants (Fig. 2).

**Fig. 2.**
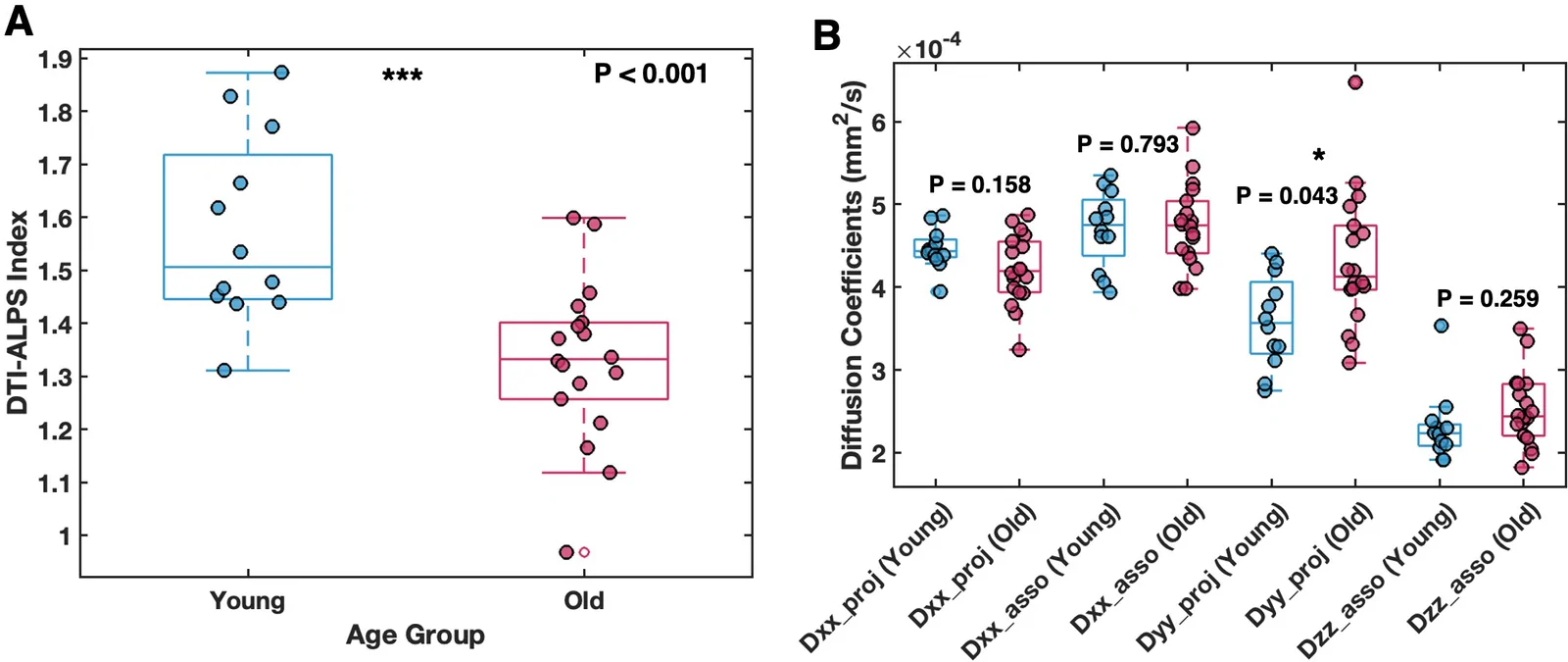
Group differences in glymphatic measures. (A) DTI-ALPS index in younger and older participants. (B) Group differences in individual diffusion coefficients. p-values in (B) are Benjamini–Hochberg false discovery rate (FDR) corrected. *p < 0.05, **p < 0.01, ***p < 0.001.

**Table 1.** Whole-brain/global multimodal MRI measures in younger and older adults.

Measure	Region	Young Mean ± SD	Old Mean ± SD	t	df	p	95% CI
DTI-ALPS index	Localized (ROI-based)	1.573 ± 0.177	1.329 ± 0.154	3.996	28	<0.001***	[0.119, 0.369]
FW fraction	White matter	0.169 ± 0.016	0.205 ± 0.033	-3.496	28	0.002**	[-0.058, -0.015]
Kw (min^-1^)	Whole brain	127.150 ± 16.270	107.639 ± 24.023	2.456	28	0.021*	[3.238, 35.784]

*p < 0.05, **p < 0.01, ***p < 0.001.

### 3.2 FW

Significantly increased white matter free water fraction was observed in older individuals compared to younger individuals (p = 0.002) (Table 1). Additionally, significant negative association (β = -3.487, p = 0.047) was observed between FW and DTI-ALPS measures across participants (Fig. 3).

**Fig. 3.**
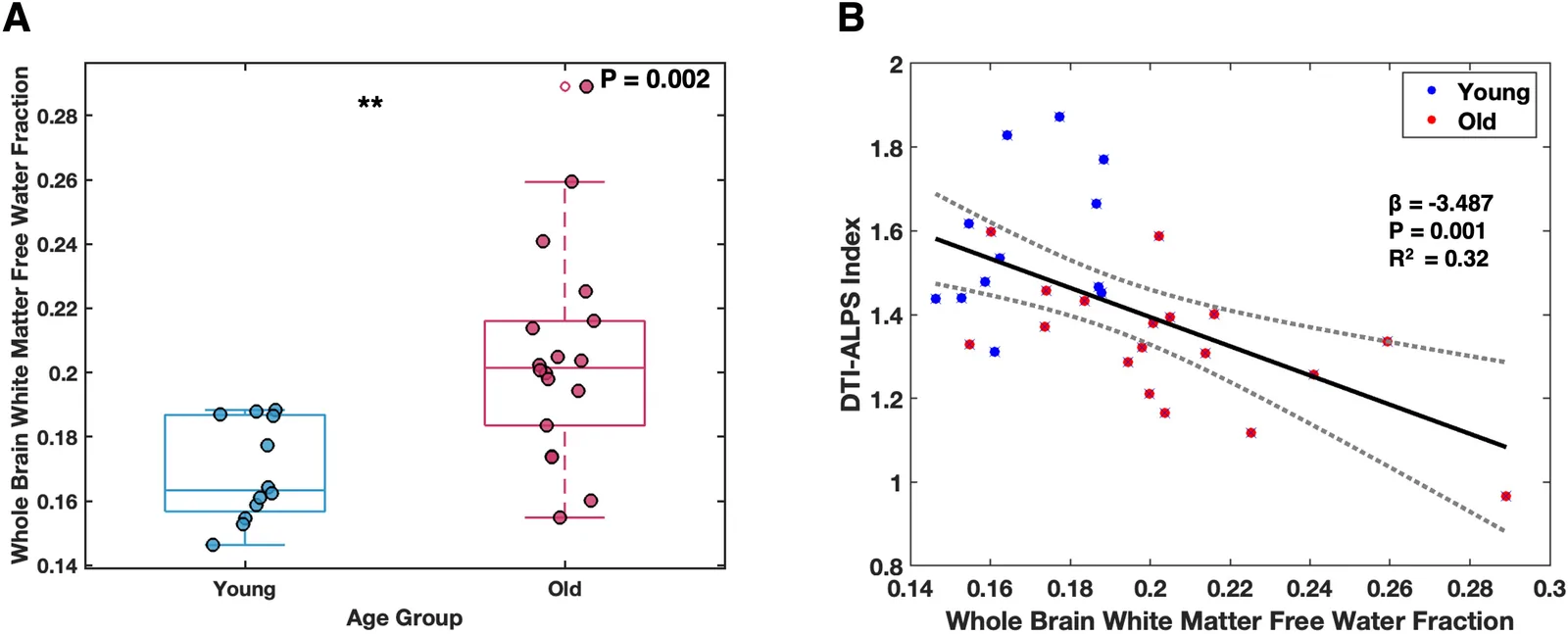
Group differences in FW measures. (A) Whole-brain white matter FW fraction in younger and older participants. (B) Association between whole-brain FW fraction and DTI-ALPS index. *p < 0.05, **p < 0.01, ***p < 0.001.

### 3.3 Kw

Significantly reduced whole-brain K_w_ measures were observed in older participants (p = 0.021) (Table 1), which negatively associated with mean FW fraction (β = -0.676 × 10^−3^, p = 0.008) and positively associated with the DTI-ALPS index (β = 0.402 × 10^−2^, p = 0.022) (Fig. 4). Older participants had also significantly reduced K_w_ in cortical gray matter (p = 0.013). Reduction in the hippocampus, entorhinal cortex, and amygdala were not statistically significant (Fig. 5, Supplementary Table S2).

**Fig. 4.**
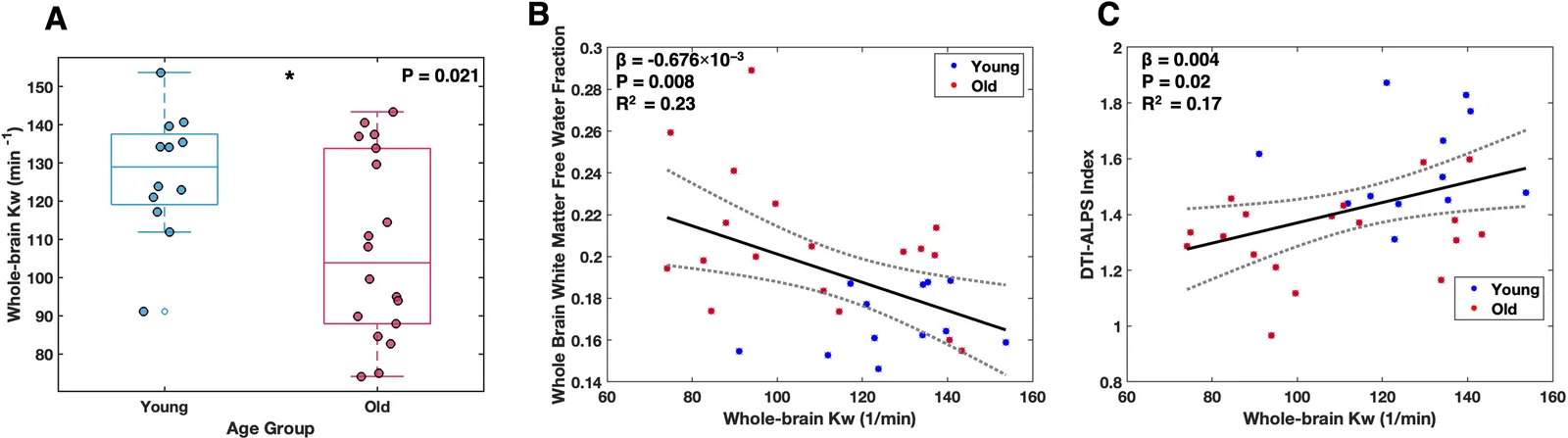
Group differences in whole-brain Kw (A) and its association with FW fraction (B) and DTI-ALPS index (C). *p < 0.05, **p < 0.01, ***p < 0.001.

**Fig. 5.**
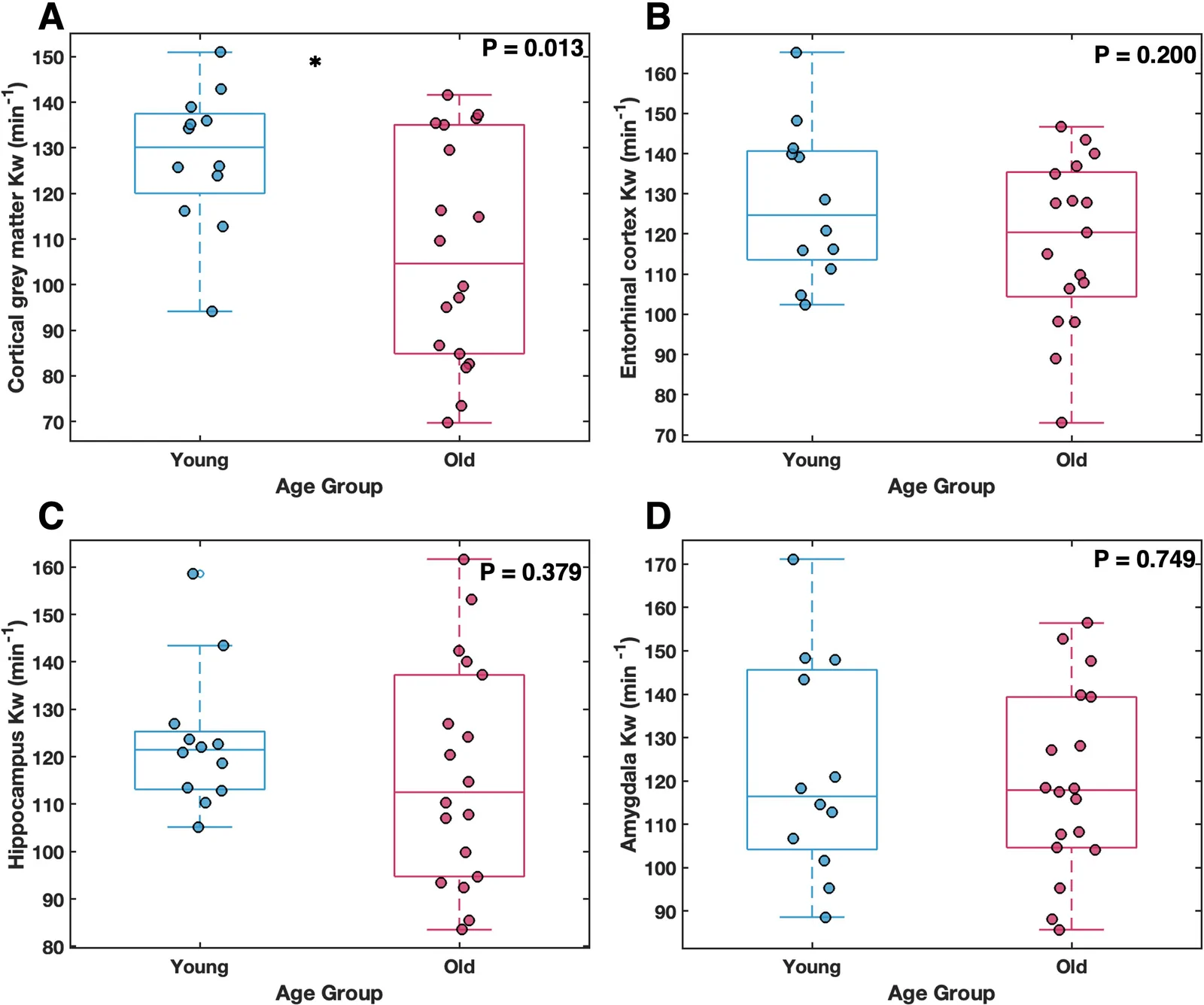
Group differences in Kw measures across grey matter regions: cortical grey matter (A), entorhinal cortex (B), hippocampus (C), and amygdala (D). *p < 0.05, **p < 0.01, ***p < 0.001.

Exploratory linear regression analyses including age group and sex as covariates indicated a significant effect of sex on FW, with higher values in males compared to females (β = 0.025, p = 0.021). No significant effects of sex were observed for ALPS (β = −0.061, p = 0.334) or Kw (β = −14.40, p = 0.075). Exploratory CBF analyses revealed a significant age-group difference, with lower CBF in the older group (p < 0.001). CBF was also significantly associated with Kw (β = 0.309, p = 0.005) (Supplementary Fig. S1). Tract-specific FW values showed significant negative associations with Kw (all p < 0.05) (Supplementary Table S1).

### 3.4 Mediation analysis

We tested whether the effect of Kw on DTI-ALPS was mediated by FW. Mediation analysis revealed a significant indirect effect of Kw on DTI-ALPS index through FW (bootstrap mean a*b = 0.0019, 95% CI [0.00014, 0.00404], p = 0.035; see Fig. 6), indicating mediation. When lateral ventricular volume (residualized for estimated total intracranial volume) was included as a covariate in the mediation model, the indirect effect was not statistically significant (a*b = 0.001560, 95% CI [-0.000259, 0.004137], p = 0.116).

**Fig. 6.**
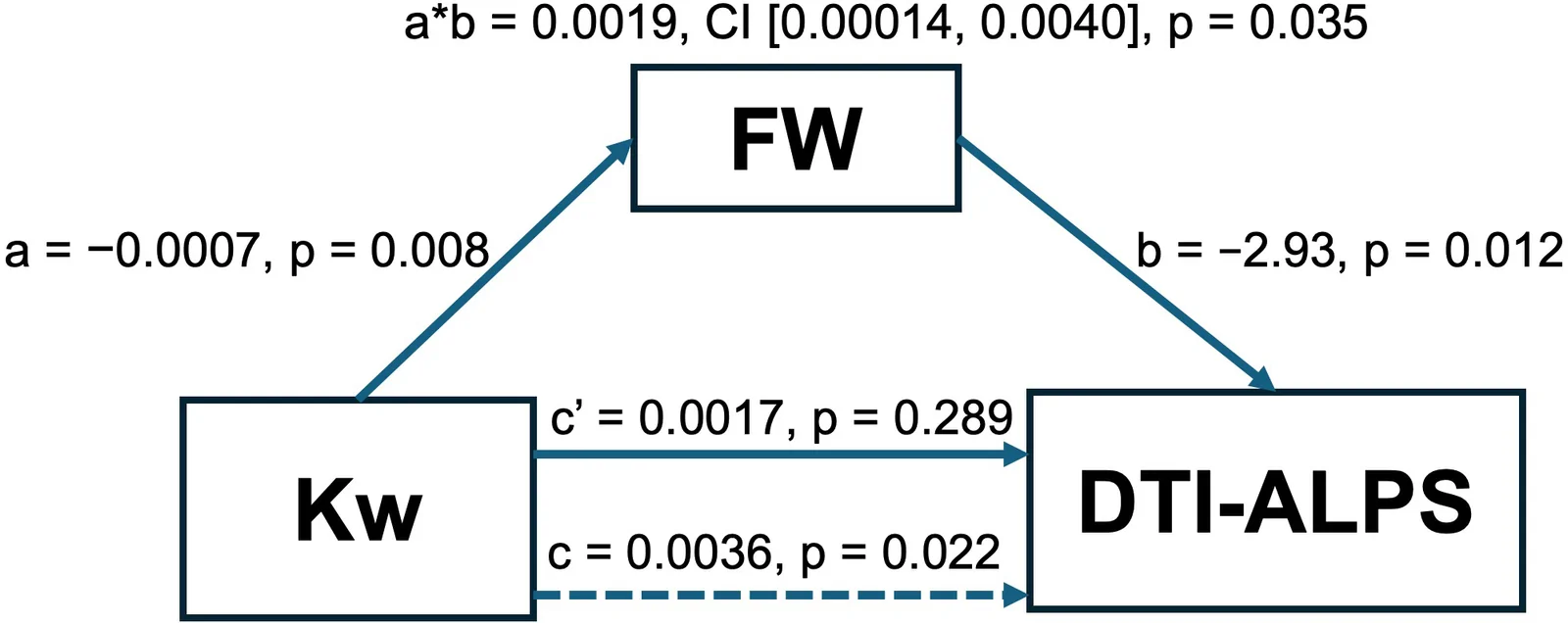
Mediation model showing the relationship between Kw and DTI-ALPS index, mediated by FW fraction. Arrows indicate the direction of effects, and coefficients represent the path from Kw to FW (a), FW to DTI-ALPS (b), the total effect (c), the direct effect (c′), and the indirect effect (a*b).

## 4 Discussion

In this study, we used multimodal MRI to investigate the association between BBB integrity and glymphatic function in healthy aging. Our study cohort included healthy younger (26 ± 3 years) and older (75 ± 7 years) individuals, representing a clear age contrast to examine differences in BBB integrity and glymphatic function. We observed age-related changes in Kw, DTI-ALPS, and FW, suggesting declining BBB function and impaired glymphatic clearance in older individuals. In older individuals, Kw and DTI-ALPS index were reduced, while white matter FW fraction was elevated. Mediation analysis further indicated that FW mediates the link between BBB integrity and glymphatic function, highlighting a potential mechanistic pathway connecting these processes.

Consistent with earlier findings, we observed a significant reduction in the DTI-ALPS index in older participants, suggesting a decline in glymphatic function with age (Han et al., 2023; Hsiao et al., 2023). This reduction was accompanied by increased white matter free water, suggesting that impaired perivascular CSF–ISF exchange may contribute to the accumulation of extracellular fluid. Reductions in whole-brain and regional Kw measures indicate subtle BBB dysfunction, which may further impede solute clearance. The trend of Kw reduction with aging is not yet fully understood. One potential explanation is dysregulation of water movement pathways due to age-related dysfunction of AQP4 (Shao et al., 2024). Subtle BBB dysfunction may permit ongoing water influx while reducing clearance efficiency, leading to increased FW fraction. This hypothesis needs to be further explored. Significant reductions in Kw may reflect impaired BBB function. Previous studies have reported sex-related differences in blood–brain barrier integrity (Shao et al., 2024) and glymphatic function (Ozsahin et al., 2024). In this study, a significant association with sex was observed for FW fraction, whereas no associations were found for ALPS or Kw. However, the present study was not designed or powered to systematically assess sex effects, and this remains an important consideration for future larger cohorts.

Our findings are consistent with earlier DP-ASL studies, which suggested a reduced Kw with aging (Morgan et al., 2024). However, Kw measured with multi-echo arterial spin labeling (ME-ASL) showed the opposite trend, increasing in older individuals. This difference in direction is not fully understood and may reflect differences in acquisition and modeling approaches between the two techniques. Dynamic contrast-enhanced MRI (DCE-MRI) is another commonly used technique to study BBB permeability (Heye et al., 2014). An earlier study comparing K_trans_ (obtained with DCE-MRI) with Kw revealed that these measures were positively associated only in the white matter, caudate, and middle cerebral artery perforator territory, but not at the whole-brain level or in grey matter regions, suggesting that they may reflect different underlying BBB mechanisms (Shao et al., 2020). Kw, which uses water as an endogenous tracer that crosses the BBB more readily than gadolinium contrast agents, also showed high test–retest reliability and reproducibility (Shao et al., 2020).

Our results indicate that BBB integrity predicts impaired glymphatic clearance, potentially mediated by extracellular white matter FW. This suggests that BBB dysfunction may increase extracellular water content, which in turn reduces glymphatic clearance efficiency. This finding is conceptually consistent with an earlier study that also examined BBB integrity (i.e., Kw) and reported a mediating role of free water for cognitive outcomes in aging (Pappas et al., 2024). Previous findings in elderly individuals reported that BBB Kw is associated with CBF, along with links between FW, enlarged perivascular spaces, and white matter hyperintensities (WMH), highlighting interactions between NVU integrity and fluid regulation (Rowsthorn et al., 2025). Consistent with these findings, our exploratory analyses showed that both CBF and tract-specific FW fraction values were associated with Kw. Our findings provide novel insights into the mechanistic role of NVU function (i.e., BBB integrity) in glymphatic clearance. Importantly, our findings highlight that subtle age-related shifts in BBB integrity and glymphatic function can occur even in the absence of disease.

FW and Kw were derived from different tissue compartments due to methodological considerations and the established reliability of each imaging approach. Although these measures do not represent a direct one-to-one tissue correspondence, they may reflect interconnected aspects of brain fluid homeostasis. White matter FW has been shown to increase with aging and reflect alterations in extracellular water and tissue microstructure (Gullett et al., 2020). BBB water exchange near neuronal cell bodies, glial cells, and neuropil may be associated with extracellular fluid levels surrounding white matter axons, providing a potential biological link between whole-brain Kw and WM FW (Pappas et al., 2024). These processes may also involve the glymphatic system, which contributes to interstitial fluid homeostasis and waste clearance in the brain (Hablitz & Nedergaard, 2021; Iliff et al., 2012). Examining the associations among whole-brain Kw, WM FW, and the ALPS index may therefore provide insights into interconnected BBB-related and fluid-regulatory processes across brain tissue types.

While DTI-ALPS is a promising surrogate marker measure of CSF flow along the perivascular spaces, its sensitivity and specificity remain debated (Ringstad, 2024). Commentaries have noted that the relationship between diffusion parameters measured at a single anatomical location and glymphatic function across the brain remains unclear, and that markers based solely on white matter water mobility may be insufficient to reflect glymphatic clearance (Piantino et al., 2023; Ringstad, 2024). It can be compromised by the selection and placement of the ROI, as well as by the resolution of the DWI data and the processing approach. In our study, ROIs were manually placed to improve anatomical accuracy. DTI-ALPS has been increasingly used as a proxy for glymphatic function, and recent improvements such as DTI-ALPS plus may enhance its sensitivity (Jiao et al., 2025). A recent contrast-enhanced glymphatic MRI (gMRI) study in a cohort with suspected CSF circulation disorders demonstrated sparse tracer enhancement in the ALPS regions, and the association between the ALPS index and gMRI markers of glymphatic function was limited (Mossige et al., 2026). These findings highlight the need for alternative techniques that are more mechanistically related to clearance and are not confined to a single global metric measured locally. Our study shows a clear distinction between younger and older DTI-ALPS measures, suggesting impaired glymphatic activity with age. Impaired glymphatic function may lead to the accumulation of metabolic byproducts over time, potentially compromising cognitive resilience (He & Sun, 2025). Sleep is known to enhance glymphatic clearance, and age-related changes in sleep may further influence CSF–ISF exchange efficiency (Ma et al., 2025; Nedergaard & Goldman, 2020; Xie et al., 2013). These results need to be further validated using improved methods for assessing CSF clearance and standardized pipelines to ensure reproducible findings.

Other approaches have been used to assess glymphatic-related function through CSF dynamics. Examples include intrathecal gadolinium-based contrast-enhanced MRI (Mossige et al., 2026), DCE-MRI (Heye et al., 2014), and perivascular space (PVS) quantification on structural MRI (Moses et al., 2023). Positron emission tomography (PET) has also been used to assess the clearance of pathological proteins, such as Aβ, in Alzheimer’s disease (Schubert et al., 2019). These approaches require exogenous tracer administration and provide complementary information about glymphatic function.

Conventionally, FW has been used to increase the sensitivity of traditional DTI metrics such as fractional anisotropy and mean diffusivity. More recently, it has gained attention as an individual surrogate marker of cerebral injury (Maillard et al., 2019). Our findings revealed significantly higher extracellular free water content in the white matter of older individuals, consistent with earlier findings, which may reflect impaired WM structural integrity in aging (Gullett et al., 2020). However, there is no direct evidence so far that excess FW content is related to neurodegeneration, which warrants further investigation. As hypothesized, we observed a significant correlation between Kw and both DTI-ALPS and FW measures, suggesting that cerebral fluid clearance mechanisms are interconnected. It is noted that in this study we focused only on WM free water content and not on gray matter structures.

Our mediation analysis suggests that impaired BBB integrity can predict glymphatic clearance through white matter FW accumulation. However, these findings were not significant when we included ventricular size as a covariate in the model. This suggests that the previously observed mediation of extracellular free water may, at least in part, reflect brain atrophy, as reflected by residualized ventricular volume. These findings may also be influenced by our relatively small sample size and should be validated in a larger cohort. Notably, BBB Kw measures were individually significantly correlated with both FW fraction and DTI-ALPS index measures, suggesting that the BBB plays a significant role in CSF clearance in the brain. Overall, our findings revealed an association between an NVU component and fluid clearance.

This study has some limitations. We used a multimodal approach to investigate BBB integrity and glymphatic clearance in healthy aging, with a cohort that had a clear age differentiation (younger: 26 ± 3 years, older: 75 ± 7 years). However, our sample size was relatively small. There were also some technical limitations. DP-ASL, used for Kw estimation, unlike DWI and T1W images, has limited spatial resolution (3.5 × 3.5 × 8 mm³), which may introduce partial volume effects in Kw measurements within smaller brain structures (Zhu et al., 2025). In this study, whole-brain Kw was the primary outcome (Shao et al., 2024), while regional analyses of aging-vulnerable structures were considered exploratory. The analyzed ROIs varied substantially in size, with large cortical grey matter containing several thousand native DP-ASL-equivalent voxels, whereas smaller structures such as the bilateral hippocampus contained approximately 78 native DP-ASL-equivalent voxels, which may increase susceptibility to partial volume effects and registration-related variability. As previously discussed, DTI-ALPS index is a proxy measurement of glymphatic function and can be influenced by ROI selection and placement, as well as by the resolution of the DWI data. DTI-ALPS metrics have been shown to be significantly influenced by white matter axonal geometry, including crossing fibers, axonal undulation, and fiber dispersion (Schilling et al., 2025). These anatomical complexities should be taken into consideration when interpreting DTI-ALPS metrics. Future work should focus on improving the sensitivity and standardization of Kw, FW, and DTI-ALPS measures in a larger cohort. In this cohort, amyloid status was not available; future studies incorporating amyloid biomarkers will be important to further elucidate whether amyloid pathology contributes to alterations in BBB integrity and glymphatic clearance.

## 5 Conclusion

In this study, we utilized multimodal MRI metrics to study BBB integrity and glymphatic clearance in healthy aging. Our findings suggest that BBB integrity predicts glymphatic activity and extracellular free water accumulation in healthy aging. Significant reductions in Kw may reflect impaired BBB function, while reduced DTI-ALPS index and increased FW fraction may indicate impaired glymphatic clearance in older individuals. FW may mediate the link between Kw and DTI-ALPS, highlighting a potential mechanistic pathway connecting these processes. The findings hold potential for advancing imaging biomarkers to preserve cognitive health in aging populations and should be validated in larger cohorts.

## Supplementary Material

Supplementary Material

## Data Availability

Data will be shared upon reasonable request for research only after ethical approval for the specific project. Code for free-water imaging is publicly available through the MarkVCID2 Biomarker Kit Protocols (https://markvcid.partners.org/markvcid2-protocols-resources).
