# Supplementary material for: Multimodal MRI insights into glymphatic clearance and blood-brain barrier integrity in healthy aging
Source: Imaging Neurosci (Camb). 2026 Oct 8;4:IMAG.a.1391. doi: 10.1162/IMAG.a.1391 (PMC13651135; doi:10.1162/IMAG.a.1391)
Supplement: Supplementary file 1 [file IMAG.a.1391_supp.pdf]

Supplementary Material

Multimodal MRI Insights into Glymphatic Clearance and Blood-Brain Barrier Integrity in Healthy Aging

Anjan Bhattarai[1,2], Yufei D. Zhu[3], Barah Albuhwailah[2], Pauline Maillard[1], Charles DeCarli[1], Audrey P. Fan[1,2]

[1] Department of Neurology, University of California, Davis, Davis, CA, USA

[2] Department of Biomedical Engineering, University of California, Davis, Davis, CA, USA

[3] Stevens Neuroimaging and Informatics Institute, University of Southern California, Los Angeles, CA, USA

Corresponding author:

Anjan Bhattarai, Department of Neurology and Department of Biomedical Engineering, University of California, Davis, 1590 Drew Avenue, Unit #100, Davis, CA 95618, USA.

Email: anbhattarai@health.ucdavis.edu

Supplementary Table 1: Exploratory tract-based analyses examining associations between regional FW and Kw. Free-water maps were generated in native diffusion space, registered to each subject's T1-weighted structural image using linear registration (FSL FLIRT), and normalized to MNI space using nonlinear registration (FSL FNIRT). Regional FW values were extracted using the JHU ICBM atlas. Tract-specific FW values and their associations with whole-brain Kw were assessed using linear regression. Reported values include regression coefficients (β), R^2, and p-values (Benjamini–Hochberg FDR corrected).

White matter tracts	β	R^2	p-value (FDR-corrected)
Anterior thalamic radiation	-0.001	0.30	0.009**
Cingulum	-0.001	0.29	0.009**
Corticospinal	-0.001	0.19	0.019*
Forceps major	-0.001	0.25	0.011*
Forceps minor	-0.001	0.21	0.019*
Inferior longitudinal fasciculus	-0.0004	0.13	0.049*
Inferior fronto-occipital fasciculus	-0.001	0.20	0.019*
Superior longitudinal fasciculus	-0.001	0.27	0.010*
Uncinate fasciculus	-0.001	0.16	0.035*

Supplementary Table 2: Regional Kw measures in younger and older adults. P-values are uncorrected and presented to illustrate potential trends.

Measure	Region	Young Mean ± SD	Old Mean ± SD	t	df	p	95%CI
**Kw (min $^{-1}$)**	Cortical grey matter	128.078 ± 15.281	107.074 ± 24.480	2.641	28	0.013*	CI [4.710, 37.298]
	Hippocampus	123.217 ± 14.771	116.400 ± 23.436	0.893	28	0.379	[-8.813, 22.446]
	Entorhinal cortex	127.816 ± 19.184	117.855 ± 20.728	1.313	27	0.200	[-5.600, 25.520]
	Amygdala	122.454 ± 24.989	119.709 ± 21.242	0.323	28	0.749	[-14.651, 20.140]

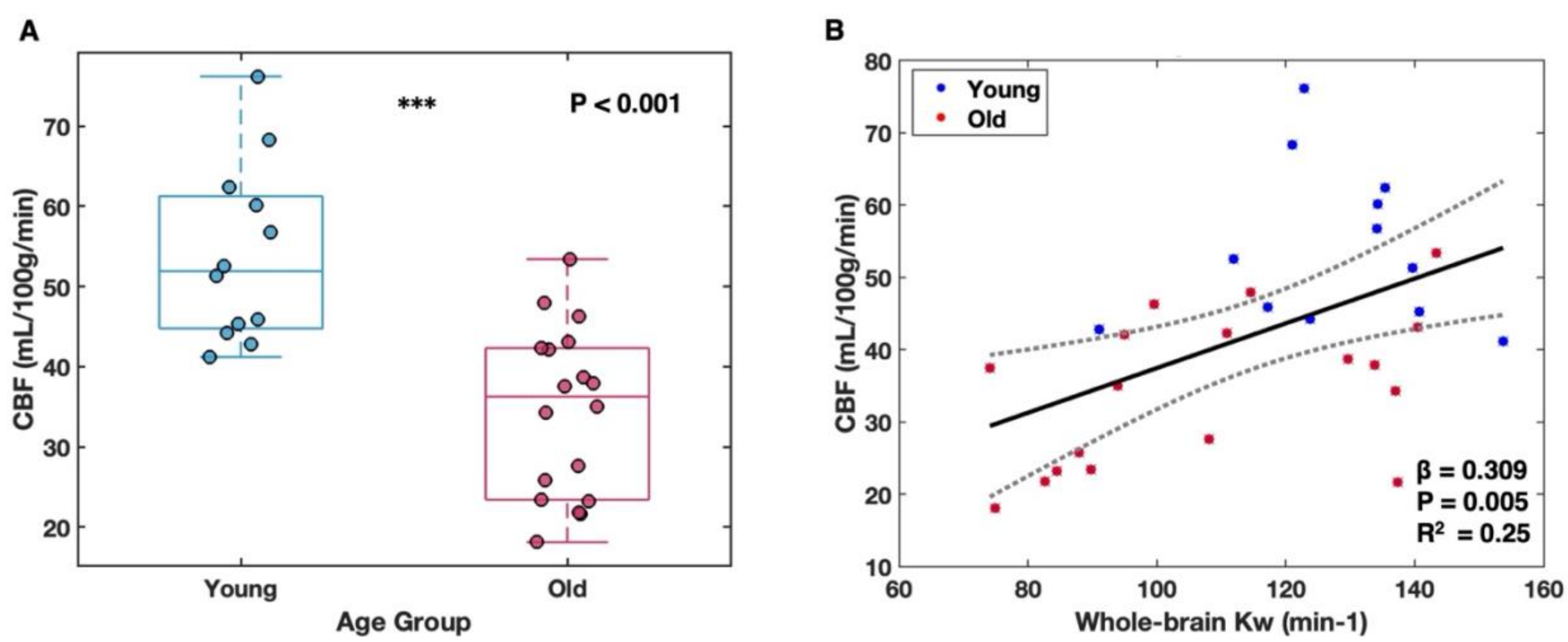

Supplementary Figure 1: Group differences in whole-brain cerebral blood flow (CBF) (A) and its association with whole-brain Kw (B).
